# Discrepancies in resistant starch and starch physicochemical properties between rice mutants similar in high amylose content

**DOI:** 10.3389/fpls.2023.1267281

**Published:** 2023-11-03

**Authors:** Mingrui Luo, Wanxin Gong, Siyan Zhang, Lanyu Xie, Yitao Shi, Dianxing Wu, Xiaoli Shu

**Affiliations:** ^1^ State Key Laboratory of Rice Biology, Key Laboratory of the Ministry of Agriculture and Rural Affairs for Nuclear Agricultural Sciences, Zhejiang University, Hangzhou, China; ^2^ Life Science and Technology Center, China National Seed Group Co., Ltd., Wuhan, China; ^3^ Hainan Institute of Zhejiang University, Yazhou Bay Science and Technology City, Sanya, China

**Keywords:** Rice (*Oryza sativa* L.), resistant starch, amylose, amylopectin, physiochemical properties

## Abstract

The content of resistant starch (RS) was considered positively correlated with the apparent amylose content (AAC). Here, we analyzed two Indica rice mutants, RS111 and Zhedagaozhi 1B, similar in high AAC and found that their RS content differed remarkably. RS111 had higher RS3 content but lower RS2 content than Zhedagaozhi 1B; correspondingly, cooked RS111 showed slower digestibility. RS111 had smaller irregular and oval starch granules when compared with Zhedagaozhi 1B and the wild type. Zhedagaozhi 1B showed a B-type starch pattern, different from RS111 and the wild type, which showed A-type starch. Meantime, RS111 had more fa and fb1 but less fb3 than Zhedagaozhi 1B. Both mutants showed decreased viscosity and swelling power when compared with the parents. RS111 had the lowest viscosity, and Zhedagaozhi 1B had the smallest swelling power. The different fine structures of amylopectin between RS111 and Zhedagaozhi 1B led to different starch types, gelatinization properties, paste viscosity, and digestibility. In addition to enhancing amylose content, modifications on amylopectin structure showed great potent in breeding rice with different RS2 and RS3 content, which could meet the increasing needs for various rice germplasms.

## Introduction

Resistant starch (RS) is a kind of starch and starch degradation products that are resistant to be digested in the small intestine but can be fermented in the large intestine by microbial flora to produce metabolites such as short-chain fatty acids (SCFAs). RS plays a beneficial role in controlling blood sugar and regulating intestinal flora (Englyst et al., 1992; Birt et al., 2013). With the development of living standards and the change in lifestyle, the number of the type 2 diabetics is increasing. High RS diet can help to prevent diabetes and reduce calorie intake, which may be helpful for weight management (Birt et al., 2013). Therefore, it is essential to develop functional foods rich in RS. According to the feature and botanical origin, RS can be classified into five subtypes, namely, RS1, RS2, RS3, RS4, and RS5. RS2 is made up of native starch granules and usually presents in raw food such as green bananas and raw potatoes (Englyst and Cummings, 1987; Faisant et al., 1995). RS3 consists of retrograded starch, mainly the recrystallized amylose, and is formed during the cooling and retrogradation of gelatinized starch and cooked foods stored at room or low temperature (Noah et al., 1998).

Rice is the staple food for more than half of the population worldwide and the primary source of nutrition and carbohydrates for many people. RS2 and RS3 is the main RS type that exists in raw and cooked rice, respectively (Ding et al., 2019). Hot cooked rice is regarded as a typically high-GI food, generally contained low RS content with less than 3% (Frei et al., 2003), which is not enough to confer the health benefits (Shu et al., 2009). Breeding rice high in RS is an effective way to improve public health. Previous studies have found that amylose content, the ratio of amylose to amylopectin and fine structure of amylopectin, had obvious impacts on RS content (Sajilata et al., 2006; Chung et al., 2011; Zhu et al., 2011). Several genes related to starch synthesis have been found to regulate RS formation in rice. *Waxy* (*Wx*), which encodes granule-bound starch synthase (GBSS) I, can regulate RS formation through impacting amylose synthesis (Jeon et al., 2010). *BEIIb* plays an important role in forming short amylopectin chains and the deficiency of *BEIIb* (*ae* mutant) in rice can significantly increase in RS content by increasing the number of B2 and B3 branched chains (Butardo et al., 2011; Abe et al., 2014; Nakata et al., 2018; Baysal et al., 2020). In addition, *SSIIIa* involved in the elongation of the B2 and B3 chains in amylopectin (Fujita et al., 2007) has been verified to be a critical gene responsible for the RS synthesis in rice, loss of function of *SSIIIa* gives rise to a high RS content in rice grain (Zhou et al., 2016). These results provided opportunities to increase RS content of rice through genetic improvement.

Up to date, enhancing amylose content is an important approach to develop high-RS rice and functional foodstuffs (Shen et al., 2022a). Some mutants or varieties with enhanced amylose content also had elevated RS, such as RS111 (Yang et al., 2006), Gaomi 2 (Kang et al., 2003), Jiangtangdao 1 (Yang et al., 2012), TRS (Wei et al., 2010), and b10 (Zhou et al., 2016). However, the palatability of rice high in RS with high amylose content were generally poor; moreover, the yield was also reduced (Tao et al., 2019; Miura et al., 2021). Thus, balancing the quality, yield, and RS content is important for high-RS rice breeding. Improving the quality of restorer and sterile lines is the key for traditional rice hybrid breeding, while the quality of sterile is majorly determined by its maintainer. In previous design breeding, we simultaneously improved restorer R7954 and maintainer II-32B, both parents of super hybrid rice IIyou7954 (II-32A×R7954), and isolated and developed two high RS mutants, RS111 (*ssIIIa* mutant) and Zhedagaozhi 1B (unknown mutant), respectively, which were similar in high amylose but different in RS properties. Clarifying discrepancies in RS and starch physicochemical properties of rice similar in high amylose content will provide some guidance for high-RS rice breeding and diversified healthy and functional foods.

## Materials and methods

### Rice materials

Elite restorer R7954 and leading commercial maintainer II-32B (Shu et al., 2007), high-RS mutants RS111, and Zhedagaozhi 1B were used in this study. RS111 and Zhedagaozhi 1B were derived, respectively, from R7954 and II-32B by irradiated with 300 Gy ^60^Co-γ rays. RS111 is an *ssIIIa* mutant (Zhou et al., 2022), while Zhedagaozhi 1B is not (data not provided) and might be a novel high AAC/RS mutant. All accessions were grown in the experiment farms of Zhejiang University (Hangzhou, China, 120.2E, 30.3N) in June 2022. The mature seed were harvested in late October 2022.

### Preparation of rice flour and starch

The rice grains were air-dried to achieve the moisture content of about 12%. The samples were dehulled using a Satake Rice Machine (Satake Co., Tokyo, Japan), milled to white rice using a Satake Rice Machine (Satake Co., Hiroshima, Japan), and then ground into flour (CT 293, Foss, Sweden) and passed through a 100-mesh sieve.

Starch was extracted according to a previous method described by Yang et al. (2006) with minor modifications. Briefly, rice flour (30 g) was suspended with 150 mL 0.2% sodium hydroxide solution and then shaken for 12h. The starch fraction was recovered by centrifugation at 4000 rpm for 10 min and repetitive scrubbed until the pH reached 7.0. The starch was dried at 40°C in a vacuum oven for 72h, passed through a 150-μm sieve and stored in a drier till used.

### Apparent amylose content, total starch, resistant starch, and lipid content

Apparent amylose content (AAC) was determined by the simplified assay as Yang et al. (2006). AAC standard samples (2%, 8%, 17%, 22%, and 28.5%) were provided by the China National Rice Research Institute. Total starch (TS) content was also measured according to the method described by Yang et al. (2006). The contents of RS2 in raw sample and RS3 in cooked sample were measured as the previous studies (Gong et al., 2021). Free lipid was measured by Hanon SOX406 fat analyzer (Hanon Group, Jinan, China) following the principle of Soxhlet extraction method [extraction solvent, petroleum ether (AR, bp, 30–60°C)].

### Scanning electron microscopy

Starch sample was directly adhered to double-sided adhesive tape mounted on an aluminum stub and coated under vacuum with platinum for 50 s (IB-5 ion coater, Eiko Co.). All coated samples were observed with a Hitachi SU8010 cold field emission SEM (Tokyo, Japan) operated at 3.0 kV.

### X-ray diffraction

X-ray diffraction pattern was performed with copper Kα radiation on an x-ray diffractometer (D8 Advance, Bruker, Germany). The scanning regions of the diffraction angle 2θ were 4°–40°, which covered most of the significant diffraction peaks of the starch crystallites, with a 0.02°-step size and a count time of 0.2 s. The crystallinity was calculated according to the method of Hayakawa et al. (1997).

### Particle size distribution

Granule size of starches was detected using a laser light scattering particle size analyzer (LS13320, Beckman Coulter, California, USA). Starch (50 mg) was mixed with 15 mL of ddH_2_O and then sonicated for 5 min. The sample was loaded to the sample port by drops until 8%–12% obscuration.

### Fourier transform infrared spectroscopy

Starch granule short-range ordered structure was detected with the Fourier transform infrared (FTIR) spectrometer (NICOLET iS50FT-IR, Thermo Fisher Scientific, USA). The scanning range was from 4,000 cm^-1^ to 400 cm^-1^ with 32 scans at 4 cm^-1^. FTIR spectra were treated in peak half-width of 19 cm^-1^ and the resolution enhancement factor of 1.9. Then the relative absorbances at 1,045, 1,022, and 995 cm^-1^ were extracted from the deconvoluted spectra and were measured from the baseline to the peak height according with Man et al. (2012).

### Amylopectin fraction and amylopectin chain-length distribution determination

Amylopectin fractionation was conducted as Kong et al. (2008). The chain-length distribution of the de-branched sample was analyzed with the Carbo-Pac PA-100 column (250 mm × 4 mm, with a guard column) using a high-performance anion-exchange chromatography system (Dionex ICS-5000+, Sunnyvale, CA, USA) coupled with a BioLC gradient pump and a pulsed amperometric detector. The samples were then eluted with a flow rate of 1 mL/min. The eluents used were A (150 mM sodium hydroxide) and B (150 mM sodium hydroxide containing 500 mM sodium acetate). A mixed elution gradient with eluent A and eluent B was set as follows: 0–10 min, 93%–82% A; 10–19 min, 82%–78% A; 19–70 min, 78%–62% A; 70–70.1 min, 62%–50% A; 70.1–85 min, 50% A; 85–85.1 min, 50%–93%. The system was stabilized by elution at 93% A for 5 min between runs.

### Thermal properties measured with DSC

Thermal properties of starch were determined by a differential scanning calorimeter (DSC) (Q20, TA Instruments, New Castle, DE, USA) equipped with DSC standard and dual sample cells. Starch (2.0 mg, dry weight basis) was weighed into an aluminum pan, and 6 μL of MilliQ water was added (Kong et al., 2015). The pan was hermetically sealed and equilibrated at room temperature for 12h, then scanned from 40°C to 11°C at a heating rate of 10°C/min with an empty sealed pan as a reference. The onset gelatinization temperature (GT) (*To*), peak temperature (*Tp*), conclusion temperature (*Tc*), and enthalpy change (*ΔH*) were analyzed with a Universal Analysis Program, Version 4.4A. The range of GT was calculated as *R* = *Tc* − *Tp*.

### Gelatinization properties in urea

Gelatinization properties was determined according to the method of Nishi et al. (2001) with minor modifications. Briefly, rice flour (10 mg) was mixed with 1 mL of 5M urea in an Eppendorf tube and incubated at 25°C for 24h. After centrifugation, sample was allowed to stand for 1h. Swelling power was calculated by subtracting the volume of supernatant from the urea solution (1 mL).

### Pasting viscosity

Pasting properties of rice flour were analyzed using a Rapid Visco Analyzer (RVA Tecmaster, Perten, Hägersten, Sweden) according to the methods described by Yang et al. (2006). The peak viscosity (PV), trough viscosity (TV), final viscosity (FV), and paste temperature (PT) were recorded; breakdown viscosity (BV = PV − TV) and setback viscosity (SV = FV − TV) were calculated.

### *In vitro* digestion study

Digestion property was studied using a standard protocol as reported by Shen et al. (2022b) with minor modifications. Rice flour of 0.5 g was mixed with 4.5 mL of water and boiled for 30 min to ensure full gelatinization. After cooling to the room temperature, the rice was homogenized for 30 s using an Ultra Turrax T25 IKA Homogenizer to mimic human chewed. After blending, crushed sample was digested through three phases (oral, gastric, and intestinal phase). During the simulated intestinal phase, totally 10 time points (0, 10, 20, 30, 45, 60, 90, 120, 150, and 180 min) was chosen for sampling. The digested starch content in the supernatant was determined by glucose analysis. Uncooked rice flour was digested in the same method above but without cooking.

The percentage of the starch hydrolysis was calculated by the following equation (1):


(1) $$SH\left(\%\right)=\frac{Sh}{Si}=\frac{Gp}{Si}\times 0.9$$

Where SH is the percentage of starch hydrolysis, Sh is the amount of hydrolyzed starch at different time points in intestinal phase, Si is the initial amount of starch in the samples, and Gp is the amount of glucose released due to starch hydrolysis. The conversion factor of 0.9 was used based on the molecular weight ratio of a starch monomer to glucose.

The experiment data were fitted to a first-order model according to equation (2), as previous reported by Goñi et al. (1997):


(2) $$C_{t}=C_{0}+C_{\infty }\left(1-e^{-kt}\right)$$

where C_t_, C_0_, and C_∞_ are the percentage of digested starch at time t, 0, and infinite time, respectively, and k is a pseudo first-order rate constant. Box Lucas model as the nonlinear curve fit model in OriginPro2022 (Origin Lab corporation, Northampton, MA, USA) was used for estimating k and C_∞_ value. Initial digestion rate in the first 20 min (IRR_20_) was calculated by equation (3) as described by Kan et al. (2020):


(3) $$IRR_{20}=\left(C_{20}-C_{0}\right)/20$$

where C_20_ and C_0_ are the percentage of digested starch at 20 min and 0 min.

### Statistical analysis

All the tests were conducted in triplicate at least. The statistics analysis was conducted using Duncan with Tukey’s multiple comparison tests following one-way analysis of variance with SPSS version 20.0 (IBM, Chicago, USA).

## Results and discussions

### Apparent amylose and resistant starch content

The AAC of R7954 and II-32B were both around 25% and significantly lower than those of their mutants. Meantime, R7954 and II-32B had similar RS content (**Table 1**). The AAC of mutant RS111 and Zhedagaozhi 1B were also similar, which were 33.85% and 34.70%, respectively, indicating that both of RS111 and Zhedagaozhi 1B were typically high amylose mutants when compared to common rice. However, obvious discrepancies in RS contents were found between two mutants. The contents of RS3 in RS111 was 4.78%, significantly higher than that in Zhedagaozhi 1B which was 2.46%, while the content of RS2 in RS111 was only 1.30%, significantly lower than that in Zhedagaozhi 1B which was as high as 17.37%. The contents of RS2 and RS3 in the flour of RS111 and Zhedagaozhi 1B were all significantly higher than those in the parents (**Table 1**). AAC showed positive correlation to both RS2 and RS3. Several high-amylose rice mutants also showed high-RS contents (Kang et al., 2003; Wei et al., 2010; Zhang et al., 2011; Yang et al., 2012; You et al., 2022).

**Table 1:** Physiochemical properties of flours and starches*.

		RS111	Zhedagaozhi 1B	R7954	II-32B
Flour	Apparent amylose (%)	33.85 ± 0.79a	34.70 ± 0.91a	25.82 ± 0.22b	25.48 ± 0.55b
	RS2 (%)	1.30 ± 0.12b	17.37 ± 0.68a	0.37 ± 0.02c	0.75 ± 0.12c
	RS3 (%)	4.78 ± 0.35a	2.46 ± 0.32b	1.39 ± 0.12c	1.27 ± 0.09c
	Swelling volume (mL)	0.139 ± 0.001c	0.066 ± 0.002d	0.233 ± 0.006a	0.153 ± 0.002b
	Lipids (%)	0.65 ± 0.02a	1.25 ± 0.01b	0.64 ± 0.03a	0.66 ± 0.01a
Starch	RS2 (%)	1.58 ± 0.10b	19.23 ± 0.49a	1.65 ± 0.12b	1.54 ± 0.07b
	RS3 (%)	3.67 ± 0.04b	4.06 ± 0.10a	1.17 ± 0.03c	1.11 ± 0.05c
	Mean diameter (μm)	6.12 ± 0.00d	7.75 ± 0.05b	8.13 ± 0.07a	6.52 ± 0.07c
	Relative crystallinity (%)	19.67 ± 0.28c	19.55 ± 0.19c	24.01 ± 0.22b	28.57 ± 0.38a
	IR ratio 1045/1022 (cm^−1^)	0.58 ± 0.00d	0.68 ± 0.02c	0.79 ± 0.02b	0.85 ± 0.02a
	IR ratio 1022/995 (cm^−1^)	1.86 ± 0.00d	1.60 ± 0.00c	1.84 ± 0.00b	1.99 ± 0.00a

*The results were expressed as mean ± SD (n = 3). The same small letters indicated that there were no significant differences among the starches or flours of rice (p< 0.05).

Considering that other components in the flour might also affect the RS content, the RS content in isolated starch were also determined. The RS2 content in isolated starch was higher than those in four for all samples. The RS3 content in the isolated starches was a little lower than those in flours for R7954, II-32B, and RS111 while higher for Zhedagaozhi 1B. Meantime, the contents of RS3 in mutant RS111 were 3.67%, lower than that in Zhedagaozhi 1B starch (4.06%). The higher RS2 in isolated starch indicated that the other components in the flour might reduce the attack of amylase to the starch granules. Although the difference in RS3 content between starch and flour might because of the existence of lipids in rice flour, Zhedagaozhi 1B had significantly higher lipids content (**Table 1**). Although it has been reported that lipid can interact with amylose to form starch-lipid complexes and contribute to the RS, the formation of starch-lipid complexes will compete with amylose retrogradation (Cui and Oates, 1999), which would impede retrogradation of the remained amylose and reduced the RS content in turn (Perera et al., 2010). Zhou et al. (2023) also found that the existence of lipids in indica rice with high amylose reduced the RS3 content.

### Morphology of starch granules

**Figure 1:**
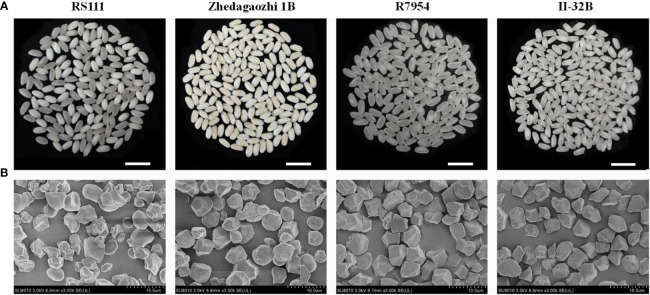
**(A)** The grain morphology of milled rice. **(B)** Scanning electron micrographs (SEM) of flour rice starch granules.

The four varieties showed obvious different morphology of grain (**Figure 1A**) and starch granules (**Figure 1B**). The starch granules of R7954 and II-32B were uniform and almost polygonal, most starch granules of Zhedagaozhi 1B also showed polyhedron, and some were nearly spherical and some with small pinholes on their surfaces, which might be caused by the unsaturated grains. Although, starch granules of RS111 were irregular and small, with some round granules (**Figure 1B**). Starch with more round and spherical granules were found to have higher RS in cooked rice, while starch with a higher percentage of polyhedral and angular granules usually have higher RS in raw rice (Ding et al., 2019; You et al., 2022). Wei et al. (2010) also found that TRS with higher RS2 had large and non-angular rounded bodies compared with its parent. That was consistent with our results that RS111 with irregular and smaller starch granules had significantly lower content of RS2. However, Zhedagaozhi 1B which had both polyhedral and oval starch granules had significant higher RS2 than all other samples, indicating other structural properties might also be responsible for the difference in RS between RS111 and Zhedagaozhi 1B.

### Crystalline structures of rice starch

**Figure 2:**
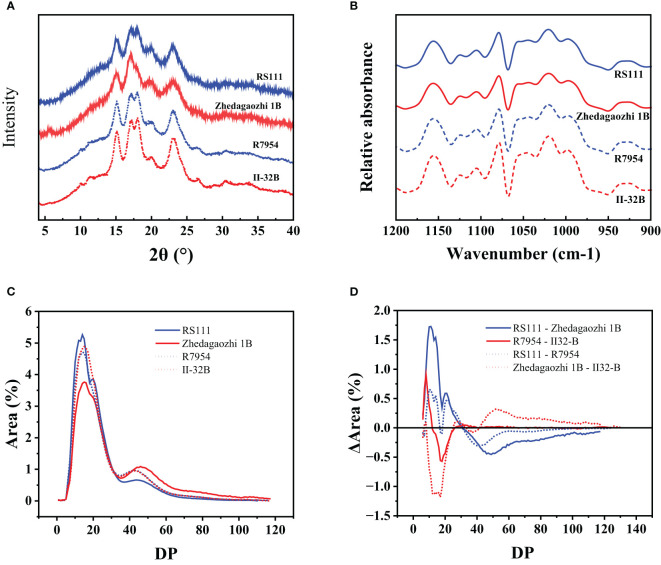
**(A)** XRD patterns of four rice starches. Data have been offset for clarify. **(B)** Fourier transform infrared (FTIR) spectroscopy spectra of four rice starches. **(C)** Chain length distribution patterns of amylopectin for four starches. **(D)** Comparison of the chain length distribution patterns of amylopectin between RS111 and Zhedagaozhi 1B.

The external chains of amylopectin interact with each other and water to form crystalline structure. The arrangement of the crystals of the granules gives rise to A, B, and C three types (Buléon et al., 1998). The x-ray diffraction pattern of mutant RS111 and two parents showed typical A-type with strong peaks at around 2θ 15.2°, 17.2°, 18°, 20°, and 23° (**Figure 2A**). However, the X-ray diffraction (XRD) pattern of Zhedagaozhi 1B showed B-type with the typical peak at 5.6° and weak peak at 18°. The relative crystallinity of mutant RS111 was 19.67%, similar with that of Zhedagaozhi 1B (19.55%), while significantly lower than those of their parents R7954 (24.01%) and II-32B (28.57%). It has been found that B-type crystalline structure could form large “blocklets” bound in crystalline layers of granules (Guo et al., 2017); the dense internal structure can enhance the resistance of digestion to a certain extent. Starch with B-type diffraction pattern (ungelatinized) have been reported to have higher levels of RS and exhibit lower starch digestibility (Magallanes-Cruz et al., 2017) than the A- and C-type starch. That can explain Zhedagaozhi 1B with B-type starch had higher RS2 (**Table 1**). Short-range ordered structure of starch granule can be observed by FT-IR, the ratio of absorption at 1,045/1,022 cm^−1^ and 1,022/995 cm^−1^ can reflect the degree of order structure and the proportion of amorphous to ordered carbohydrate structure in starch granule external region, respectively (Sevenou et al., 2002). All four varieties showed similar FTIR profiles without new peaks appeared (**Figure 2B**). RS111 and Zhedagaozhi 1B showed lower 1,045/1,022 cm^−1^ ratio than their parents respectively (**Table 1**), indicating that there were less short-range order helical structures in the external region of two mutants. Meantime, RS111 and Zhedagaozhi 1B also showed different short-range ordered structure, RS111 had higher ratio of 1,045/1,022 cm^−1^ and lower ratio of 1,022/995 cm^−1^ than Zhedagaozhi 1B (**Table 1**).

AAC had been confirmed to be negatively correlated with the degree of particle surface order (Sun et al., 2022) and RC positively related to the degree of particle surface ordering (Zou et al., 2022). The lower 1,045/1,022 cm^−1^ in both mutants reflected as the lower crystallinity and consistent with their higher AAC (**Table 1**). However, RS111 and Zhedagaozhi 1B had similar AAC and similar RC; the different short-range ordered structure might be due to the different fine structure of amylopectin, as in addition to AAC, fine amylopectin structure also influenced the ordered structure greatly.

### Chain-length distributions of amylopectin

Based on the DP, the side chains of amylopectin can be classified into fa (DP ≤ 12), fb1 (13 ≤ DP ≤ 24), fb2 (25 ≤ DP ≤ 36), and fb3 (DP ≥ 37), corresponding to fractions A, B1, B2, and B3 and longer chains, respectively (Hanashiro et al., 1996). R7954 had more proportions of fa and less fb1 than II-32B, but the proportions of fb2 and fb3 were similar. RS111 had significantly higher proportion of fa and fb1 but lower of fb3 than Zhedagaozhi 1B (**Table 2**), with significant increases in DP ≤ 30 (**Figure 2D**). Although, the differences in chain length distribution between R7954 and II-32B were obviously smaller than those between RS111 and Zhedagaozhi 1B (**Figure 2D**). When compared with their parents, RS111 had significant increases in fa, fb1 and decrease in fb3, while Zhedagaozhi 1B had significantly decreased proportions of fa, fb1 and fb2, but increased fb3 (**Table 2**). Meantime, Chain-length distributions (CLDs) of amylopectin showed a peak DP (degree of polymerization) at 14 and 15 for RS111 and Zhedagaozhi 1B, respectively. Zhedagaozhi 1B showed significantly different CLDs from RS111, R7954, and II-32B.

**Table 2:** Branch chain-length distribution of rice starch amylopectins*.

	Peak DP	fa	fb1	fb2	fb3	fa/fb1	fa/fb2	fa/fb3
		DP6-12(%)	DP13-24(%)	DP25-36(%)	DP ≥ 37(%)			
RS111	14	21.77 ± 0.18a	48.56 ± 0.26a	14.65 ± 0.34a	14.91 ± 0.74c	0.45	1.49	1.46
Zhedagaozhi 1B	15	13.22 ± 0.17d	39.20 ± 0.06c	14.25 ± 0.31a	33.19 ± 0.05a	0.34	0.93	0.40
R7954	14	18.89 ± 0.60b	44.68 ± 0.03b	14.31 ± 0.60a	22.01 ± 0.03b	0.42	1.32	0.86
II-32B	15	16.61 ± 0.49c	48.70 ± 0.59a	13.44 ± 0.51a	21.14 ± 0.61b	0.34	1.24	0.79

*The results were expressed as mean ± SD (n = 3). The same small letters indicated that there were no significant differences among the starches or flours of rice (p< 0.05).

The differential CLDs between RS111 and Zhedagaozhi 1B might explain the differential short ordered structure (**Table 1**), which might be responsible majorly by the amylopectin structure. The proportion of fa, fb1 showed negative impacts on RS2, while the proportion of fb3 showed positive correlation with RS2. Ramadoss et al. (2018) also found RS2 content showed significantly positive correlation with medium and long chains (DP > 12). Amylopectin with intermediate and longer can pack into intact or dense structures, which showed stronger resistance to enzymatic hydrolysis (Chi et al., 2021). However, the chain length distribution did not show direct correlation with RS3, Zhedagaozhi 1B with the lowest fa and fb1 still had higher RS3 content, inconsistent with our previous reports that RS3 showed significantly positive correlation with fa (Shu et al., 2007). That might, because RS3 was formed majorly after retrogradation, the ordered hierarchical starch structures were progressively disrupted during cooking. The reassembled structures involving amylose–amylose, amylose–lipids, and amylopectin–amylose during cooking, cooling might be more important for RS3 content (Chi et al., 2021), especially for Zhedagaozhi 1B.

### Thermal characteristics of rice flour and starch

Thermal properties of native starches and flours were summarized in **Table 3**. The onset temperature (*To*), peak GT (*Tp*), conclusion temperature (*Tc*), and enthalpy of gelatinization (*ΔH*_gel_) varied significantly among four materials. The *To*, *Tp*, *Tc*, and *ΔH*_gel_ of Zhedagaozhi 1B were higher than those of RS111 for both starch and flour, which was consistent with the difference between their parents. When compared with their parents, RS111 had lower *To*, *Tp*, *Tc*, and *ΔH*_gel_, while Zhedagaozhi 1B had higher *To*, *Tp*, *Tc*, and *ΔH*_gel_. Meantime, the value of *R*(*Tc*-*To*)of mutant Zhedagaozhi 1B was significantly higher than other varieties, and the differences of thermal characteristics between starch and flour were more significant for Zhedagaozhi 1B, which may greatly be influenced by the non-starch components such as lipids. GT has been found to be negatively correlated with DP6-11 and positively correlated with DP12-24 (Bao et al., 2009). RS111 with the highest proportion of fa had the lowest GT while Zhedagaozhi 1B had the lowest proportion of fa had the highest GT (**Tables 2**, **3**). Furthermore, Zhedagaozhi 1B with the highest GT had the highest content of RS2 while RS111 with the lowest GT had the highest RS3, in parts line with previous study that DSC thermal parameters showed a negative correlation with RS in cooked rice and retrogradation rice but positive correlation with RS in raw milled rice (You et al., 2022).

**Table 3:** Thermal properties of four rice *.

		*To* (°C)	*Tp* (°C)	*Tc* (°C)	*△Hgel* (J/g)	*R* (°C)
Flour	RS111	57.19 ± 0.18c	63.81 ± 0.07d	71.06 ± 0.15d	7.60 ± 0.31b	13.87 ± 0.33b
	Zhedagaozhi 1B	70.31 ± 0.85a	80.94 ± 0.11a	88.54 ± 0.30a	9.00 ± 0.44a	18.23 ± 1.08a
	R7954	59.27 ± 0.04b	65.71 ± 0.14c	73.58 ± 0.25c	9.52 ± 0.05a	14.31 ± 0.21b
	II-32B	70.05 ± 0.07a	75.04 ± 0.24b	79.71 ± 0.21b	9.55 ± 0.22a	9.65 ± 0.22c
Starch	RS111	58.55 ± 0.13d	63.89 ± 0.26d	69.68 ± 0.21d	10.43 ± 0.35c	11.13 ± 0.32b
	Zhedagaozhi 1B	63.33 ± 0.39b	77.64 ± 0.13a	89.51 ± 0.24a	16.33 ± 1.47a	26.19 ± 0.49a
	R7954	61.94 ± 0.07c	65.49 ± 0.13c	71.05 ± 0.31c	13.55 ± 0.21b	9.11 ± 0.26c
	II-32B	68.79 ± 0.78a	73.77 ± 0.25b	79.58 ± 0.35b	15.83 ± 0.56a	10.80 ± 0.54b

*The results were expressed as mean ± SD (n = 3). To, onset temperature; Tp, peak temperature; Tc, conclusion temperature; ΔH_gel_, enthalpy of gelatinization; R, Tc − To.

Thermal parameters can reflect the double helix unfolding and crystallite fusion in the starch crystalline region during the gelatinization process of starch. *ΔH* primarily reflected the loss of double helical order (Li et al., 2017). The highest GT and *ΔH* of II32-B indicated the gelatinization of II32-B needed more energy, which might result from its lower proportion of amorphous to ordered carbohydrate structure in starch granule external region (**Table 1**). The lower thermal temperature and *ΔH* in RS111 may be majorly due to its higher proportion of fa (**Table 2** and **Figure 2D**), lower RC, and higher proportion of amorphous to ordered carbohydrate structure (**Table 1**).

### Gelatinization properties of rice starch

**Figure 3:**
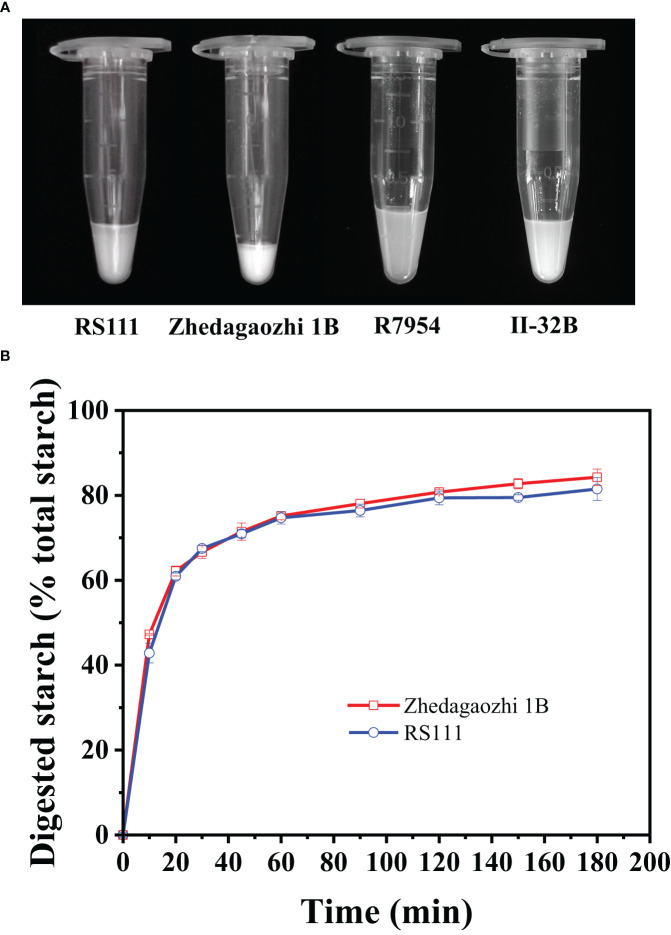
**(A)** Effects of 5M urea on the swelling of starch granules from the endosperm of two genotypes. **(B)** *In-vitro* starch hydrolysis curves of the two rice varieties cooked in different forms. Error bars represent the standard deviation of the mean of triplicate digestion.

When gelatinized in 5 M urea, four varieties showed different degrees of gelatinization (**Figure 3A**). R7954 and II-32B swelled obviously in urea, whereas the starch of RS111 and Zhedagaozhi 1B were hardly gelatinized, especially Zhedagaozhi 1B, which was scarcely gelatinized. Meanwhile, the solubility of Zhedagaozhi 1B determined by the volume of sediment was significantly lower than that of RS111 (**Table 1**). These results were consistent with the chain length distribution of amylopectin that starch with more short chain was easier to be gelatinized (Li, 2022). The lowest swelling and gelatinizing of Zhedaogaozhi 1B might explain for its highest RS2 content as swelling power showed negative correlation with RS (Singh et al., 2010). However, Zhedaogaozhi 1B had less RS3 content than RS111, although it is hard to be gelatinized in urea, that might be because of the gelatinization in urea cannot reflect completely the gelatinization in boiling water and the reassembled structures during cooking and cooling played more important role in RS3 formation (Chi et al., 2021).

### Pasting properties of rice flours and starches

**Figure 4:**
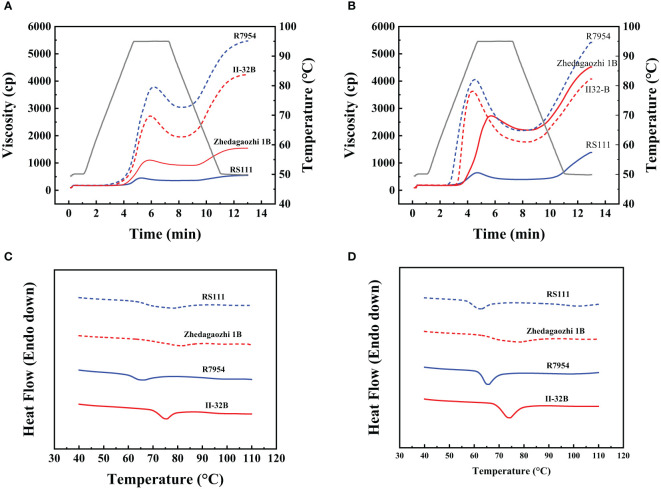
**(A)** Pasting properties of rice flours measured by Rapid Visco Analyzer (RVA). **(B)** Pasting properties of rice starches measured by RVA. **(C)** Thermal properties of the four rice fours measured by DSC. **(D)** Thermal properties of the four rice starches measured by DSC.

The RVA profiles showed significant differences among four rice varieties (**Figures 4A, B**). All RVA parameters, namely, PV, TV, breakdown viscosity, FV, and setback viscosity of Zhedagaozhi 1B were dramatically higher than that of RS111, both in starch and flour (**Table 4**). While the pasting temperature (PT) of RS1111 flour was higher than that of Zhedagaozhi 1B, and PT showed significantly positive correlation with RS3 (**Table 3**). Srichuwong and Jane (2007) found that starches consisting of amylopectin with more short A chains (DP 6–12) had a lower PT and PV and larger breakdown value since the short branch chains could not provide strong interaction to maintain the integrity of the swollen granules. The current results were partially consistent with this study, suggesting that there were other factors affected the pasting properties of rice starch between two mutants.

**Table 4:** Pasting viscosity of four rice *.

		PV (cP)	TV (cP)	BV (cP)	FV (cP)	SV (cP)	PT (°C)
Flour	RS111	452.7 ± 5.5d	361.7 ± 5.7d	91.0 ± 2.0b	547.7 ± 4.0d	186.0 ± 8.7d	92.3 ± 0.8a
	Zhedagaozhi 1B	1115.5 ± 14.8c	923.5 ± 16.3c	192.0 ± 1.4b	1540.0 ± 2.8c	616.5 ± 19.1c	87.1 ± 0.6b
	R7954	3811.5 ± 38.9a	3033.0 ± 11.3a	778.5 ± 50.2a	5539.5 ± 96.9a	2506.5 ± 108.2a	81.1 ± 0.0c
	II-32B	2749. 0 ± 89.1b	2009.0 ± 59.3b	740.0 ± 52.3a	4221.3 ± 36.7b	2212.3 ± 56.9b	79.2 ± 1.2c
Starch	RS111	623.7 ± 20.0d	388.3 ± 7.6c	235.3 ± 12.7c	1424.7 ± 39.0d	1036.3 ± 42.0c	80.0 ± 1.0a
	Zhedagaozhi 1B	2746.7 ± 25.4c	2230.7 ± 21.4a	516.0 ± 17.3b	4573.3 ± 49.8b	2355.3 ± 45.0b	80.0 ± 0.5a
	R7954	4009.0 ± 66.5a	2226.0 ± 53.7a	1783.0 ± 120.2a	5362.0 ± 80.6a	3136.0 ± 134.4a	69.6 ± 0.5c
	II-32B	3615.0 ± 17.0b	1823.5 ± 74.2b	1791.5 ± 91.2a	4210.5 ± 187.4c	2387.0 ± 113.1b	76.4 ± 1.1b

*The results were expressed as mean ± SD (n = 3). The same small letters indicated that there were no significant differences among the starches or flours of rice (p< 0.05). PV, peak viscosity; TV, trough viscosity; BV, breakdown viscosity; FV, final viscosity; SV, setback viscosity; PT, pasting temperature.

### Digestion properties

Cooked flours were homogenized to mimic chewing and then digested using the standard INFOGEST2.0 method (Brodkorb et al., 2019). The digestion data of two mutants both showed good fit with a first-order equation (*R* ^2^ > 0.86). RS111 showed a lower digestion extent than that of Zhedagaozhi 1B (**Figure 4B** and **Table 5**). The digestion extent (C_∞_) and the digestion rate (k) of RS111 was significantly lower than that of Zhedagaozhi 1B (**Table 5**). Considering the calculated values of C_∞_ were affected by the digestion time, the experimentally determined values of C_120_ were also presented, which were similar as C_∞_ (**Table 5**). Although Holm et al. (1988) found that the degree of starch gelatinization is an important determinant for the rate of starch hydrolysis *in vitro* (Holm et al., 1988) and Zhedagaozhi 1B was scarcely gelatinized, gelatinized and retrograded starch can benefit the formation of RS, which has been reported to be decisive for the glycemic index of rice (Kumar et al., 2018). The RS3 content should be the major contributor for the digestion rate in cooked rice; thus, RS111 with higher RS3 content had slower digestion rate (**Tables 1**, **5**).

**Table 5:** Estimated kinetic parameters for starch digestion of the two rice varieties*.

	C_∞_ (%)	C_120_ (%)	k (min^−1^)	IRR_10_ (% min^−1^)
**RS111**	77.8 ± 0.2**	77.7 ± 0.2**	0.074 ± 0.003	4.07 ± 0.11
**Zhedagaozhi 1B**	79.1 ± 0.1**	79.1 ± 0.1**	0.077 ± 0.002	4.24 ± 0.06

*The results were expressed as mean ± SD (n = 3). The “**” indicates significant correlations at the 0.01 levels (2-tailed).

## Conclusions

RS111 and Zhedagaozhi 1B had similar AAC with around 34%, significantly higher than their wild-type parent and common rice. Although two wild types had similar properties, RS111 and Zhedagaozhi 1B conferred different physiochemical properties, such as viscosity, gelatinization parameters, swelling power, and digestibility. RS111 had higher RS3 content but lower RS2 content than Zhedagaozhi 1B; correspondingly, cooked RS111 showed slower digestibility. Differences in physiochemical properties might be due to the different starch structure between RS111 and Zhedagaozhi 1B. The B-type starch granule and high proportion of fb3 in Zhedagaozhi 1B may be responsible for its high-RS3 content, while the smaller irregular and oval starch granules, high proportion of fa in RS111 might be the major contributor to its rich RS3. As RS111 and Zhedagaozhi 1B are derived from restorer and maintainer, respectively; they can be used directly as new rice varieties, or as parents for high-RS hybrid rice breeding through improving other restorers and maintainers. Fine structure of amylopectin seemed very important for determining the RS type and content when amylose content is similar. Modifications on amylopectin structure showed great potential in breeding rice with different RS2 and RS3 content, which could meet the increasingly needs for various rice germplasms.

## Data availability statement

The raw data supporting the conclusions of this article will be made available by the authors, without undue reservation.

## Author contributions

ML: Methodology, Data curation, Investigation, Validation, Writing – original draft. WG: Data curation, Formal Analysis, Methodology, Writing – original draft, Visualization. SZ: Investigation, Data curation, Methodology, Writing – original draft. LX: Data curation, Investigation, Writing – original draft. YS: Data curation, Investigation, Writing – original draft. DW: Conceptualization, Funding acquisition, Project administration, Supervision, Validation, Writing – review & editing. XS: Formal Analysis, Methodology, Visualization, Writing – review & editing, Funding acquisition.

## References

[B1] Abe N. Asai H. Yago H. Oitome N. F. Itoh R. Crofts N.. (2014). Relationships between starch synthase I and branching enzyme isozymes determined using double mutant rice lines. BMC Plant Biol. 14, 80. doi: 10.1186/1471-2229-14-80 24670252 PMC3976638

[B2] Bao J. Xiao P. Hiratsuka M. Sun M. Umenoto T. (2009). Granule-bound SSIIa protein content and its relationship with amylopectin structure and gelatinization temperature of rice starch. Starch/Stärke 61, 431–437. doi: 10.1002/star.200800115

[B3] Baysal C. He W. Drapal M. Villorbina G. Medina V. Capell T.. (2020). Inactivation of rice starch branching enzyme IIb triggers broad and unexpected changes in metabolism by transcriptional reprogramming. Proc. Natl. Acad. Sci. U. S. A. 117, 26503–26512. doi: 10.1073/pnas.2014860117 33020297 PMC7584904

[B4] Birt D. F. Boylston T. Hendrich S. Jane J. L. Hollis J. Li L.. (2013). Resistant starch: Promise for improving human health. Adv. Nutr. 4, 587–601. doi: 10.3945/an.113.004325 24228189 PMC3823506

[B5] Brodkorb A. Egger L. Alminger M. Alvito P. Assunção R. Ballance S.. (2019). INFOGEST static in *vitro* simulation of gastrointestinal food digestion. Nat. Protoc. 14, 991–1014. doi: 10.1038/s41596-018-0119-1 30886367

[B6] Buléon A. Colonna P. Planchot V. Ball S. (1998). Starch granules: Structure and biosynthesis. Int. J. Biol. Macromolecules 23, 85–112. doi: 10.1016/S0141-8130(98)00040-3 9730163

[B7] Butardo V. M. Fitzgerald M. A. Bird A. R. Gidley M. J. Flanagan B. M. Larroque O.. (2011). Impact of down-regulation of starch branching enzyme IIb in rice by artificial microRNA-and hairpin RNA-mediated RNA silencing. J. Exp. Bot. 62, 4927–4941. doi: 10.1093/jxb/err188 21791436 PMC3193005

[B9] Chi C. Li X. Huang S. Chen L. Zhang Y. Li L.. (2021). Basic principles in starch multi-scale structuration to mitigate digestibility: A review. Trends Food Sci. Technol. 109, 154–168. doi: 10.1016/j.tifs.2021.01.024

[B10] Chung H. J. Liu Q. Lee L. Wei D. (2011). Relationship between the structure, physicochemical properties and in *vitro* digestibility of rice starches with different amylose contents. Food Hydrocolloids 25, 968–975. doi: 10.1016/j.foodhyd.2010.09.011

[B11] Cui R. Oates C. G. (1999). The effect of amylose-lipid complex formation on enzyme susceptibility of sago starch. Food Chem. 65, 417–425. doi: 10.1016/S0308-8146(97)00174-X

[B12] Ding Y. Huang J. Zhang N. Rasmussen S. K. Wu D. Shu X. (2019). Physiochemical properties of rice with contrasting resistant starch content. J. Cereal Sci. 89, 102815. doi: 10.1016/j.jcs.2019.102815

[B13] Englyst H. N. Cummings J. H. (1987). Digestion of polysaccharides of potato in the small intestine of man. Am. J. Clin. Nutr. 45, 423–431. doi: 10.1093/ajcn/45.2.423 3812341

[B14] Englyst H. N. Kingman S. M. Cummings J. H. (1992). Classification and measurement of nutritionally important starch fractions. Eur. J. Clin. Nutr. 46 Suppl 2, S33–S50. 1330528

[B15] Faisant N. Buléon A. Colonna P. Molis C. Lartigue S. Galmiche J. P.. (1995). Digestion of raw banana starch in the small intestine of healthy humans: structural features of resistant starch. Br. J. Nutr. 73, 111–123. doi: 10.1079/BJN19950013 7857906

[B16] Frei M. Siddhuraju P. Becker K. (2003). Studies on the in *vitro* starch digestibility and the glycemic index of six different indigenous rice cultivars from the Philippines. Food Chem. 83, 395–402. doi: 10.1016/S0308-8146(03)00101-8

[B17] Fujita N. Yoshida M. Kondo T. Saito K. Utsumi Y. Tokunaga T.. (2007). Characterization of SSIIIa-deficient mutants of rice: The function of SSIIIa and pleiotropic effects by SSIIIa deficiency in the rice endosperm. Plant Physiol. 144, 2009–2023. doi: 10.1104/pp.107.102533 17586688 PMC1949899

[B18] Gong W. Liu T. Zhou Z. Wu D. Shu X. Xiong H. (2021). Physicochemical characterizations of starches isolated from Tetrastigma hemsleyanum Diels et Gilg. Int. J. Biol. Macromolecules 183, 1540–1547. doi: 10.1016/j.ijbiomac.2021.05.117 34019925

[B19] Goñi I. Garcia-Alonso A. Saura-Calixto F. (1997). A starch hydrolysis procedure to estimate glycemic index. Nutr. Res. 17, 427–437. doi: 10.1016/S0271-5317(97)00010-9

[B20] Hanashiro I. Abe J. I. Hizukuri S. (1996). A periodic distribution of the chain length of amylopectin as revealed by high-performance anion-exchange chromatography. Carbohydr. Res. 283, 151–159. doi: 10.1016/0008-6215(95)00408-4

[B21] Hayakawa K. Tanaka K. Nakamura T. Endo S. Hoshino T. (1997). Quality characteristics of waxy hexaploid wheat (Triticum aestivum L.): Properties of starch gelatinization and retrogradation. Cereal Chem. 74, 576–580. doi: 10.1094/CCHEM.1997.74.5.576

[B22] Holm J. Lundquist I. Bjork I. Eliasson A. C. Asp N. G. (1988). Degree of starch gelatinization, digestion rate of starch in *vitro*, and metabolic response in rats. Am. J. Clin. Nutr. 47, 1010–1016. doi: 10.1093/ajcn/47.6.1010 3287891

[B23] Jeon J. S. Ryoo N. Hahn T. R. Walia H. Nakamura Y. (2010). Starch biosynthesis in cereal endosperm. Plant Physiol. Biochem. 48, 383–392. doi: 10.1016/j.plaphy.2010.03.006 20400324

[B24] Kan L. Oliviero T. Verkerk R. Fogliano V. Capuano E. (2020). Interaction of bread and berry polyphenols affects starch digestibility and polyphenols bio-accessibility. J. Funct. Foods 68, 103924. doi: 10.1016/j.jff.2020.103924

[B25] Kang H. J. Hwang I. K. Kim K. S. Choi H. C. (2003). Comparative structure and physicochemical properties of Ilpumbyeo, a high-quality japonica rice, and its mutant, Suweon 464. J. Agric. Food Chemisty 51, 6598–6603. doi: 10.1021/jf0344946 14558783

[B26] Kong X. Bertoft E. Bao J. Corke H. (2008). Molecular structure of amylopectin from amaranth starch and its effect on physicochemical properties. Int. J. Biol. Macromolecules 43, 377–382. doi: 10.1016/j.ijbiomac.2008.07.018 18755216

[B27] Kong X. Zhu P. Sui Z. Bao J. (2015). Physicochemical properties of starches from diverse rice cultivars varying in apparent amylose content and gelatinisation temperature combinations. Food Chem. 172, 433–440. doi: 10.1016/j.foodchem.2014.09.085 25442575

[B28] Sajilata M. G. Singhal R. S. Kulkarni P. R. (2006). Resistant starch - A review. Compr. Rev. Food Sci. Food Saf. 5, 1–17. doi: 10.1111/j.1541-4337.2006.tb00076.x 33412740

[B29] Kumar A. Sahoo U. Baisakha B. Okpani O. A. Ngangkham U. Parameswaran C.. (2018). Resistant starch could be decisive in determining the glycemic index of rice cultivars. J. Cereal Sci. 79, 348–353. doi: 10.1016/j.jcs.2017.11.013

[B30] Li C. (2022). Recent progress in understanding starch gelatinization - An important property determining food quality. Carbohydr. Polymers 293, 119735. doi: 10.1016/j.carbpol.2022.119735 35798430

[B31] Li P. He X. Dhital S. Zhang B. Huang Q. (2017). Structural and physicochemical properties of granular starches after treatment with debranching enzyme. Carbohydr. Polymers 169, 351–356. doi: 10.1016/j.carbpol.2017.04.036 28504155

[B32] Magallanes-Cruz P. A. Flores-Silva P. C. Bello-Perez L. A. (2017). Starch structure influences its digestibility: A review. J. Food Sci. 82, 2016–2023. doi: 10.1111/1750-3841.13809 28753728

[B33] Man J.-M. Cai C.-H. Yan Q.-X. Hu M.-Z. Liu Q.-Q. Wei C.-X. (2012). Applications of infrared spectroscopy in the analysis of ordered structure of starch grain. Acta Agronomica Sin. 38, 505–513. doi: 10.3724/sp.j.1006.2012.00505

[B34] Miura S. Koyama N. Crofts N. Hosaka Y. Abe M. Fujita N. (2021). Generation and *starch characterization of non-tran*sgenic BEI and BEIIb Double Mutant rice (*Oryza sativa*) with ultra-high level of resistant starch. Rice 14, 3. doi: 10.1186/s12284-020-00441-0 33409744 PMC7788159

[B35] Nakata M. Miyashita T. Kimura R. Nakata Y. Takagi H. Kuroda M.. (2018). MutMapPlus identified novel mutant alleles of a rice starch branching enzyme IIb gene for fine-tuning of cooked rice texture. Plant Biotechnol. J. 16, 111–123. doi: 10.1111/pbi.12753 28499068 PMC5785365

[B36] Nishi A. Nakamura Y. Tanaka N. Satoh H. (2001). Biochemical and genetic analysis of the effects of amylose-extender mutation in rice endosperm. Plant Physiol. 127, 459–472. doi: 10.1104/pp.010127 11598221 PMC125082

[B37] Noah L. Guillon F. Bouchet B. Buléon A. Molis C. Gratas M.. (1998). Digestion of Carbohydrate from White beans (*Phaseolus vulgaris* L.) in healthy humans. J. Nutr. 128, 977–985. doi: 10.1093/jn/128.6.977 9614157

[B38] Perera A. Meda V. Tyler R. T. (2010). Resistant starch: A review of analytical protocols for determining resistant starch and of factors affecting the resistant starch content of foods. Food Res. Int. 43, 1959–1974. doi: 10.1016/j.foodres.2010.06.003

[B39] Sevenou O. Hill S. E. Farhat I. A. Mitchell J. R. (2002). Organisation of the external region of the starch granule as determined by infrared spectroscopy. Int. J. Biol. Macromolecules 31, 79–85. doi: 10.1016/S0141-8130(02)00067-3 12559430

[B40] Shen L. Li J. Li Y. (2022a). Resistant starch formation in rice: Genetic regulation and beyond. Plant Commun. 3, 100329. doi: 10.1016/j.xplc.2022.100329 35576157 PMC9251435

[B41] Shen Y. Gong W. Li Y. Deng J. Shu X. Wu D.. (2022b). The physiochemical and nutritional properties of high endosperm lipids rice mutants under artificially accelerated ageing. LWT 154, 112730. doi: 10.1016/j.lwt.2021.112730

[B42] Shu X. Jia L. Gao J. Song Y. Zhao H. Nakamura Y.. (2007). The influences of chain length of amylopectin on resistant starch in rice (Oryza sativa L.). Starch/Staerke 59, 504–509. doi: 10.1002/star.200700640

[B43] Shu X. Jia L. Ye H. Li C. Wu D. (2009). Slow digestion properties of rice different in resistant starch. J. Agric. Food Chem. 57, 7552–7559. doi: 10.1021/jf900988h 20349922

[B44] Singh J. Dartois A. Kaur L. (2010). Starch digestibility in food matrix: a review. Trends Food Sci. Technol. 21, 168–180. doi: 10.1016/j.tifs.2009.12.001

[B45] Srichuwong S. Jane J.-L. (2007). Physicochemical properties of starch affected by molecular composition and structures: A review. Food Sci. Biotechnol. 16 663–674.

[B46] Sun H. Fan J. Tian Z. Ma L. Meng Y. Yang Z.. (2022). Effects of treatment methods on the formation of resistant starch in purple sweet potato. Food Chem. 367, 130580. doi: 10.1016/j.foodchem.2021.130580 34371274

[B47] Tao K. Yu W. Prakash S. Gilbert R. G. (2019). High-amylose rice: Starch molecular structural features controlling cooked rice texture and preference. Carbohydr. Polymers 219, 251–260. doi: 10.1016/j.carbpol.2019.05.031 31151523

[B48] Wei C. Qin F. Zhou W. Yu H. Xu B. Chen C.. (2010). Granule structure and distribution of allomorphs in C-type high-amylose rice starch granule modified by antisense RNA inhibition of starch branching enzyme. J. Agric. Food Chem. 58, 11946–11954. doi: 10.1021/jf103412d 21033746

[B49] Yang C. Z. Shu X. L. Zhang L. L. Wang X. Y. Zhao H. J. Ma C. X.. (2006). Starch properties of mutant rice high in resistant starch. J. Agric. Food Chem. 54, 523–528. doi: 10.1021/jf0524123 16417315

[B50] Yang R. Sun C. Bai J. Lou Z. Shi B. Zhang J.. (2012). A putative gene sbe3-rs for resistant starch mutated from SBE3 for starch branching enzyme in rice (Oryza sativa L.). PloS One 7, e43026. doi: 10.1371/journal.pone.0043026 22937009 PMC3427327

[B51] You H. Liang C. Zhang O. Xu H. Xu L. Chen Y.. (2022). Variation of resistant starch content in different processing types and their starch granules properties in rice. Carbohydr. Polymers 276, 118742. doi: 10.1016/j.carbpol.2021.118742 34823776

[B52] Zhang G. Y. Cheng Z. J. Zhang X. Guo X. P. Su N. Jiang L.. (2011). Double repression of soluble starch synthase genes SSIIa and SSIIIa in rice (Oryza sativa L.) uncovers interactive effects on the physicochemical properties of starch. Genome 54, 448–459. doi: 10.1139/g11-010 21595523

[B53] Zhou Y. Cheng Z. Jiang S. Cen J. Wu D. Shu X. (2022). High temperature boosts resistant starch content by altering starch structure and lipid content in rice ssIIIa mutants. Front. Plant Sci. 13. doi: 10.3389/fpls.2022.1059749 PMC9715984 36466223

[B54] Zhou Y. Cheng Z. JIang S. Cen J. Yuan S. Yu C.. (2023). Inactivation of SSIIIa enhances the RS content through altering starch structure and accumulating C18:2 in japonica rice. Carbohydr. Polymers 318, 121141. doi: 10.1016/j.carbpol.2023.121141 37479448

[B55] Zhu L. J. Liu Q. Q. Wilson J. D. Gu M. H. Shi Y. C. (2011). Digestibility and physicochemical properties of rice (Oryza sativa L.) flours and starches differing in amylose content. Carbohydr. Polymers 86, 1751–1759. doi: 10.1016/j.carbpol.2011.07.017

[B56] Zou J. Li Y. Su X. Wang F. Li Q. Xia H. (2022). Structure and processing properties of nine yam (Dioscorea opposita thunb) starches from South China: A comparison study. Molecules 27, 2254. doi: 10.3390/molecules27072254 35408653 PMC9000772

